# RNAi-mediated *CHS-2* silencing affects the synthesis of chitin and the formation of the peritrophic membrane in the midgut of *Aedes albopictus* larvae

**DOI:** 10.1186/s13071-023-05865-3

**Published:** 2023-08-02

**Authors:** Chen Zhang, Yanjuan Ding, Min Zhou, Ya Tang, Rufei Chen, Yanrong Chen, Yating Wen, Shigui Wang

**Affiliations:** Hangzhou Normal University, Hangzhou, China

**Keywords:** *Aedes albopictus*, *CHS-2*, Chitin synthesis, RNA interference, Peritrophic membrane

## Abstract

**Background:**

Mosquitoes are an important vector of viral transmission, and due to the complexity of the pathogens they transmit, vector control may be the most effective strategy to control mosquito-borne diseases. Chitin is required for insect growth and development and is absent in higher animals and plants, so regulating the chitin synthesis pathway can serve as a potentially effective means to control vector insects. Most of the current research on the *chitin synthase* (*CHS*) gene is focused on *chitin synthase-1* (*CHS-1*), while relatively little is known about *chitin synthase-2* (*CHS-2*).

**Results:**

The *CHS-2* gene of *Ae. albopictus* is highly conserved and closely related to that of *Aedes aegypti*. The expression of *CHS-2* in the third-instar larvae and pupal stage of *Ae. albopictus* was relatively high, and *CHS-2* expression in adult mosquitoes reached the highest value 24 h after blood-feeding. In the fourth-instar larvae of *Ae. albopictus*, *CHS-2* expression was significantly higher in the midgut than in the epidermis. Silencing *CHS-2* in *Ae. albopictus* larvae had no effect on larval survival and emergence. The expression of four genes related to chitin synthesis enzymes was significantly upregulated, the expression level of three genes was unchanged, and only the expression level of *GFAT* was significantly downregulated. The expression of chitin metabolism-related genes was also upregulated after silencing. The level of chitin in the midgut of *Ae. albopictus* larvae was significantly decreased, while the chitinase activity was unchanged. The epithelium of the midgut showed vacuolization, cell invagination and partial cell rupture, and the structure of the peritrophic membrane was destroyed or even absent.

**Methods:**

The expression of CHS-2 in different developmental stages and tissues of Aedes albopictus was detected by real-time fluorescence quantitative PCR (qPCR). After silencing CHS-2 of the fourth-instar larvae of Ae. albopictus by RNA interference (RNAi), the expression levels of genes related to chitin metabolism, chitin content and chitinase activity in the larvae were detected. The structure of peritrophic membrane in the midgut of the fourth-instar larvae after silencing was observed by paraffin section and hematoxylin–eosin (HE) staining.

**Conclusion:**

*CHS-2* can affect midgut chitin synthesis and breakdown by regulating chitin metabolic pathway-related genes and is involved in the formation of the midgut peritrophic membrane in *Ae. albopictus*, playing an important role in growth and development. It may be a potential target for enhancing other control methods.

**Graphical Abstract:**

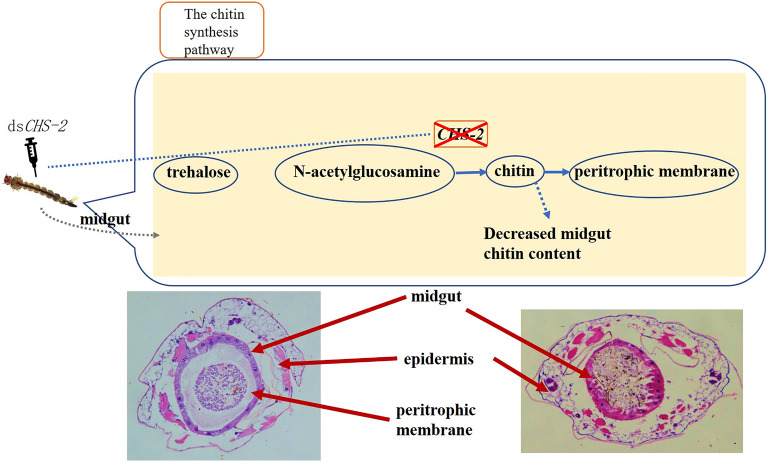

## Background

Mosquitoes feed on the blood of various animals and humans and transmit viruses that cause many diseases, such as dengue, malaria, chikungunya, and yellow fever. Several species of mosquitoes have a strong invasive capacity [1]. At present, mosquito-borne diseases are a great global public health challenge [2, 3]. Due to the complexity of mosquito-borne pathogens, eradication of vectors has become one of the more effective strategies for controlling infectious diseases. At present, the main method for mosquito control is the use of chemical insecticides, such as pyrethroids [4]. However, these insecticides can affect non-target organisms [5], and it has been shown that mosquitoes have already developed resistance to chemical insecticides [6, 7]. Therefore, there is an urgent need to find new eco-friendly insecticidal methods. Currently, the alternatives mainly include plant-derived insecticides [8, 9], genetically modified mosquitoes [10–12], and insecticidal microorganisms [13, 14].

Chitin is a linear polysaccharide polymer composed of multiple *N*-acetylglucosamines connected by β-1,4 glycosidic bonds. It is currently the second-largest known biopolymerized polysaccharide in the world, second only to cellulose [15]. It is present in the shell of invertebrates and the cell wall of fungi, and mainly plays a role in support, defence, and water loss prevention [16]. In insects, chitin is found mainly on the epidermis, trachea, and peritrophic membrane [17]. The midgut is the main digestive organ of insects, and it is also the entry point for a variety of pathogens in addition to its central role in the digestion and absorption of nutrients. The peritrophic membrane is a noncellular semipermeable structure surrounding the midgut that can protect the midgut from mechanical damage and can also block the entry of microbial pathogens and macromolecular aggregates. It is the first line of defence in the insect midgut [18]. However, in pest control efforts, it is also an obstacle for the entry of insecticides and insecticidal microorganisms [19]. Therefore, how to destroy the peritrophic membrane and make the insecticidal substances more effective has become a research focus [20–22]. The main components of the peritrophic membrane are protein and chitin. Previous studies have shown that chitin plays an indispensable role in the barrier of the peritrophic membrane [23]. Moreover, mammals and plants do not contain chitin [24], so the synthesis of chitin and the formation of peritrophic membranes have become potential targets for pest control. Specific control of chitin synthesis and degradation has become a new approach for pest control [25, 26], in view of the effect of simple use of inhibitors of chitin synthesis such as benzophenylurea (BPU) or other chemical compounds on non-target organisms such as bees [27].

The synthesis of chitin is an extremely complex process. Existing studies have shown that the raw material for chitin synthesis in insects is trehalose [28], which transforms into chitin through the action of trehalase (TRE), hexokinase (HK), glucose-6-phosphate isomerase (G6PI), glutamine–fructose-6-phosphate aminotransferase (GFAT), glucosamine-6-phosphate-*n*-acetyltransferase (GNPNA), phosphoacetylglucosamine mutase (PAGM), uridine diphosphate (UDP)-*N*-acetylglucosamine pyrophosphorylase (UAP), and chitin synthetase (CHS) [29].

The most important enzymes in the chitin synthesis pathway are TRE at the initial step and CHS at the final step [30]. CHS is a member of the glycosyltransferase family and is a large transmembrane protein with a molecular weight of 160–180 kD [31]. A comparison of CHS in various insects showed that CHS contained three conserved domains: domains A, B, and C. Domain A is located at the N end and is a transmembrane helix, domain B is the active centre of CHS, and domain C is located in terminal C and contains seven transmembrane helices [32]. Most insects contain two kinds of CHS, chitin synthetase A (CHSA) and chitin synthetase B (CHSB), which are encoded by *CHS-1* and *CHS-2*, respectively [33]. Several studies have shown that the two genes are involved in the synthesis of chitin in different insect body parts. *CHS-1* is mainly involved in chitin synthesis in the epidermis and trachea, while *CHS-2* is highly expressed only in insect midgut epithelial cells and is responsible for synthesizing chitin in the peritrophic membrane [34]. Since Tellam et al. first cloned the full-length complementary DNA (cDNA) sequence of the *CHS* gene of *Lucilia cuprina* [32], the *CHS* sequences of *Drosophila melanogaster* [35], *Tribolium castaneum* [36], *Spodoptera exigua* [37], *Anopheles gambiae* [38], *Leptinotarsa decemlineata* [39], and other insects have been cloned. There have been several studies on *CHS* as a potential target for pest control [40, 41], but these studies mainly focused on *CHS-1*; for example, after *CHS-1* gene expression levels were decreased in *Culex pipiens pallens* larvae and pupae, the moulting, pupation, and eclosion of the larvae were affected [42]. Guo et al. confirmed that *CHS-1* was involved in the development of drug resistance in *Cx. pipiens pallens* [43]. Huang et al. investigated interference with the normal expression of *CHS-1* in *Diaphorina citri* by RNA interference (RNAi), which caused the mortality and deformity rate to increase significantly and the moulting rate to decrease [44]. Zhang et al. demonstrated that after interference with *Locusta migratoria CHS-1* and its two variants, migratory locusts showed increased mortality and moulting rates [45]. Mohammed et al. studied knockdown of the *CHS-1* gene of *Phthorimaea operculella*, and larvae exhibited difficulty moulting and increased mortality [46].

*Aedes albopictus*, also known as the Asian tiger mosquito, belongs to the order Diptera, and is an important viral vector that can spread many pathogens such as dengue fever and yellow fever [47]. *Aedes albopictus* has a strong reproductive ability, and its eggs have strong drought resistance and vitality, can be stored for a long time in a dry environment, and can be easily carried and spread [48].

In this study, we analysed the expression of *CHS-2* in *Ae. albopictus*, used RNAi technology to silence the *CHS-2* gene in *Ae. albopictus*, and combined histological observation and biochemical analysis to study the function of the *CHS-2* gene at the messenger RNA (mRNA) level and enzyme activity level and explore the possibility of *CHS-2* as a potential target for controlling the *Ae. albopictus* population.

## Methods

### Mosquitos

*Aedes albopictus* were provided by Sun Yat-sen University (Guangdong Province, China), and were fed and stably passaged for more than three generations in an artificial climate chamber at 27 ± 1 °C with 70% relative humidity and light/dark (L:D) = 14:10. Cat food and yeast powder were ground into a mixed powder at a 2:1 ratio for feeding first- and second-instar larvae. The third- and fourth-instar larvae were fed cat chow powder, and post-eclosion adults were fed 10% sugar water. Mice were used as blood samples for female mosquitoes to induce egg-laying, following the method of Hsu et al. [49].

### Cloning and analysis of *CHS-2* in *Ae. albopictus*

Total RNA was extracted from the fourth-instar larvae of *Ae. albopictus* using the TRIzol method. The quality and concentration of RNA were detected by agarose gel electrophoresis and microspectrophotometry (Thermo Fisher, USA). cDNA was synthesized according to the instructions of the Prime Script™ RT Reagent Kit (Haofeng, Hangzhou, China). The sequence of the *CHS-2* gene (LOC109405983) of *Ae. albopictus* was found using the National Center for Biotechnology Information (NCBI) database, and specific primers (Table 1) were designed using Primer Premier 5.0 software to amplify intermediate sequence fragments with the cDNA template obtained in the polymerase chain reaction (PCR) system. The reaction mixture included cDNA (1 μl), forward and reverse primers (1 μl), dNTP (2.5 mM, 2 μl), 10× buffer (Mg^2+^, 2.5 μl), 5 U/μl Ex Taq (0.2 μl), and double-distilled water (ddH_2_O) (17.3 μl). PCR was performed using the following conditions: 5 min at 95 °C; 30 s at 95 °C, 30 s at 58 °C, and 1 min at 72 °C, for a total of 30 cycles, and then 10 min at 72 °C. The PCR products were separated and identified by electrophoresis on a 1% agarose gel, and the target bands were recovered by a DiaSpin PCR Product Purification Kit (Sangon Biotech, Shanghai, China), ligated into the pMD™ 18-T vector (Haofeng, Hangzhou, China), and transformed into *Escherichia coli* DH5α. After ampicillin screening, positive clones were selected and sequenced after screening with ampicillin (Shangya, Hangzhou, China). Phylogenetic trees were constructed using the neighbor-joining method in MEGA 5.0, with Jones–Taylor–Thornton as the model. Evolutionary distances were calculated by Poisson correction [50].

**Table 1:** Primers for amplification of the *CHS-2* intermediate fragment

Primer name	Forward primer (5′-3′)	Reverse primer (5′-3′)
*CHS-2*	AGTCAACGCACCGTACAGGA	GAAGAGCAACACCAAACCGA

### Determination of spatio-temporal expression patterns of the *CHS-2* gene in *Ae. albopictus*

The normally raised *Ae. albopictus* eggs (12 h), third- and fourth-instar larvae, pupae, adult *Ae. albopictus* (female) at 24 h and 48 h after eclosion, and adult *Ae. albopictus* (female) at 24 h and 48 h after blood-sucking were collected. At the same time, the midgut and epidermis of the fourth-instar larvae were collected by dissection in 1× phosphate-buffered solution (PBS). Larvae and tissues from 300 mg were collected from each group for the experiments. RNA was extracted, and cDNA was synthesized for real-time fluorescence-based quantitative PCR (qPCR). The qPCR reaction mixture (10 µl) contained 5 µl of TB Green^®^ Premix Ex Taq™, 3.2 µl of ddH_2_O, and 0.4 µl each of upstream and downstream primers and 1 µl cDNA. The qPCR procedure was as follows: 10 min at 94 °C; 30 s at 94 °C, 30 s at 59 °C, 45 s at 72 °C for a total of 30 cycles, and then 10 min at 72 °C. The default dissolution curve analysis was used. The *actin* gene (NCBI: DQ657949.1) was used as an internal control [51], and the 2^−△△CT^ method was used to calculate the relative expression of *CHS-2* in the tissues of *Ae. albopictus* at each developmental stage and fourth-instar larvae.

### Microinjection

The successfully sequenced *CHS-2* fragment was used as a template, and T7 promoter sequences were added to the 5′ end of *CHS-2* primers (Table 2). Double-stranded RNA (dsRNA) was synthesized using a T7 RiboMAX™ Express RNAi System kit (LABOOT, Hangzhou, China) and purified. The same method was used to synthesize dsRNA) of the green fluorescent protein (*GFP*) gene as a negative control. Well-developed *Ae. albopictus* fourth-instar larvae of the same size were randomly selected. Two hundred nanograms of ds*CHS-2* was dissolved in 100 nl of water and injected into the soft spot between the penultimate and third-to-last body segments of larvae using a microinjection device, and the same amount of ds*GFP* was injected as a negative control. Three biological replicates were set up, and 150 larvae were injected in each replicate. The injected larvae were reared normally. Larval mortality was recorded at 24 h and 48 h after injection, and the rates of pupation and emergence of these pupae were recorded within 72 h after injection. Fluorescence qPCR was used to detect the relative expression of the *CHS-2* gene in larvae at 24 h and 48 h after injection. The reaction mixture was the same as above.

**Table 2:** Primers for synthesis of dsRNA

Primer name	Forward primer (5′-3′)	Reverse primer (5′-3′)
ds*CHS-2*	GGATCCTAATACGACTCACTATAGGAGTCAACGCACCGTACAGGA	GGATCCTAATACGACTCACTATAGGGAAGAGCAACACCAAACCGA
ds*GFP*	GGATCCTAATACGACTCACTATAGGAAGGGCGAGGAGCTGTTCACCG	GGATCCTAATACGACTCACTATAGGCAGCAGGACCATGTGATCGCGC

The underlined is the T7 promoter

### Evaluation of chitin synthesis-related genes

Ten fourth-instar larvae were randomly selected 24 h and 48 h after injection of ds*CHS-2* and ds*GFP*. RNA was extracted and cDNA was synthesized. Through NCBI and related literature, the genes related to chitin synthesis and chitin metabolizing enzymes were selected, including *TRE1* (NCBI: XM_029860981.1), *TRE2* (NCBI: XM_0196760980), *HK* (NCBI: AY705876.1), *G6PI* (NCBI: XM_019673634.3), *GFAT* (NCBI: XM_019671518.2), *GNPNA* (NCBI: XM_019707567.2), *PAGM* (NCBI: XM_019671844.2), *UAP* (NCBI: GAPW01001510.1), *Cht2* (NCBI: XM_029879282.1), and *Cht10* (NCBI: XM_029869372.1). Primer Premier 5.0 software was used to design qPCR primers (Table 3), and the expression level was measured by qPCR. The reaction system and procedure were the same as above. Fourth-instar larvae 24 h after injection of ds*CHS-2* and ds*GFP* were selected again and dissected. The level of chitin in the midgut of larvae was determined according to the method reported by Liu et al. [52], and the principle is that chitinase hydrolyses chitin to produce *N*-acetylglucosamine, and the intermediate compound produced by the cross-reaction of *N*-acetylglucosamine and alkali can further react with *p*-dimethylaminobenzaldehyde to produce chromogenic substances. Chitinase activity in the midgut of *Ae. albopictus* larvae was evaluated using a chitinase kit (Jiancheng, Nanjing, China). The midgut protein concentration was measured using a BCA protein concentration assay kit (Takara, Japan), and the specific experimental steps were in accordance with the instructions. Tissues were collected from 45 larvae in each group for the experiments.

**Table 3:** Primers for qPCR

Primer name	Forward primer (5′-3′)	Reverse primer (5′-3′)	GenBank
*Actin*	GCTACGTCGCCCTGCACTT	AGGAACGACGGCTGGAAGA	DQ657949.1
*CHS-2*	GGAGACCAAAGGATGGGACG	CCTGTAAGGACGATGACGAATGT	XM_019679038.3
*Cht2*	GACTCGGATGACAAGGGTTT	CAGGGCTTCACATACTTCGTT	XM_029879282.1
*Cht10*	GCCACGGATACTTAGAATAGCG	CTGTTTGACGGTCGTTGATTT	XM_029869372.1
*HK*	GGGAGAAGCCAGCGAAGATA	GATGTCTGCTTCGGGATGTG	AY705876.1
*PAGM*	GCCATTTCTGACATGCTACT	CGTTTCGGTCTTCTACTTTGA	XM_019671844.2
*UAP*	AGTGCTCTATTTACACGCTCAT	ACTCCGACCGCTTCATTT	GAPW01001510.1
*GNPNA*	CAGTCCTCCCATTTCAGCC	AACGAAACATCGCCCACC	XM_019707567.2
*GFAT*	TCAACGGGCAACATCCAG	AAGCGTCCGATGCAAAGA	XM_019671518.2
*G6PI*	GGAGGATGACATTCGCTTCG	CGGTGATACGGTTCTTGGAG	XM_019673634.3
*TRE1*	GGACTGAACAGCCAGGAAGC	CGTTAGCCGGTCGCCATA	XM_029860981.1
*TRE2*	ATGGACGCCGTGGAGACA	CGCAATAGTAATCCTGCTTGTCTT	XM_0196760980

### Observation of the peritrophic membrane of the midgut

Fourth-instar larvae 24 h and 48 h after injection of dsRNA were used as materials, and the head and tail were removed according to the Gregor et al. production, sectioning, and staining method [53], fixed with formaldehyde, washed with gradient concentrations of ethanol, and then washed with xylene to make them transparent. The larvae were embedded in paraffin with an embedding machine (MICROM, Germany) and sectioned into 5-µm-thick sections. After deparaffinization with xylene, the samples were cleaned with gradient concentrations of ethanol and double-distilled water. The samples were stained with hematoxylin–eosin (HE) staining. After adding coverslips to the slides, a microscope camera system (Axio Observer A1 + Stemi2000, ZEISS, Germany) was used to photograph and observe.

### Data analysis

Excel was used to organize the data, and IBM SPSS Statistics 23.0 and one-way analysis of variance (ANOVA) with Duncan’s test were used for significance analysis (significant differences indicated as *P < 0.05, **P < 0.01). SigmaPlot 10.0 was used for the plot. All experiments were set up with three biological replicates and three technical replicates.

## Results

### Cloning and sequence analysis of the *CHS-2* gene of *Ae. albopictus*

**Fig. 1:**
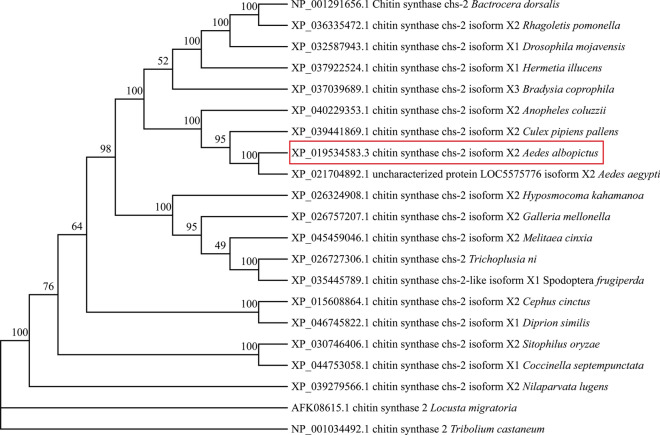
For *Ae. albopictus CHS-2* phylogenetic tree analysis. The red box is the position of *Ae. albopictus* in the phylogenetic tree, using the ortho-linked method and using Jones–Taylor–Thornton as the model. Evolutionary distances were calculated by Poisson correction

The full-length *CHS-2* gene sequence of *Ae. albopictus* (NCBI: XM_019679038.3) was obtained by searching the NCBI database. The full length of the *CHS-2* gene was 6206 base pairs (bp), and we amplified a 463-bp-long fragment of the gene starting at 766 bp and ending at 1229 bp for dsRNA synthesis. An evolutionary tree was constructed by selecting the *CHS-2* amino acid sequences of 20 species of insects with similar homology to *Ae. albopictus*, including *Aedes aegypti*, *L. migratoria manilensis*, *Culex quinquefasciatus*, *D. melanogaster*, *Tribolium subtilis*, and *Diprionidae*, which are relatively close homologues to *Ae. albopictus* (Fig. 1).

### Spatio-temporal expression pattern of *Ae. albopictus CHS-2* gene

**Fig. 2:**
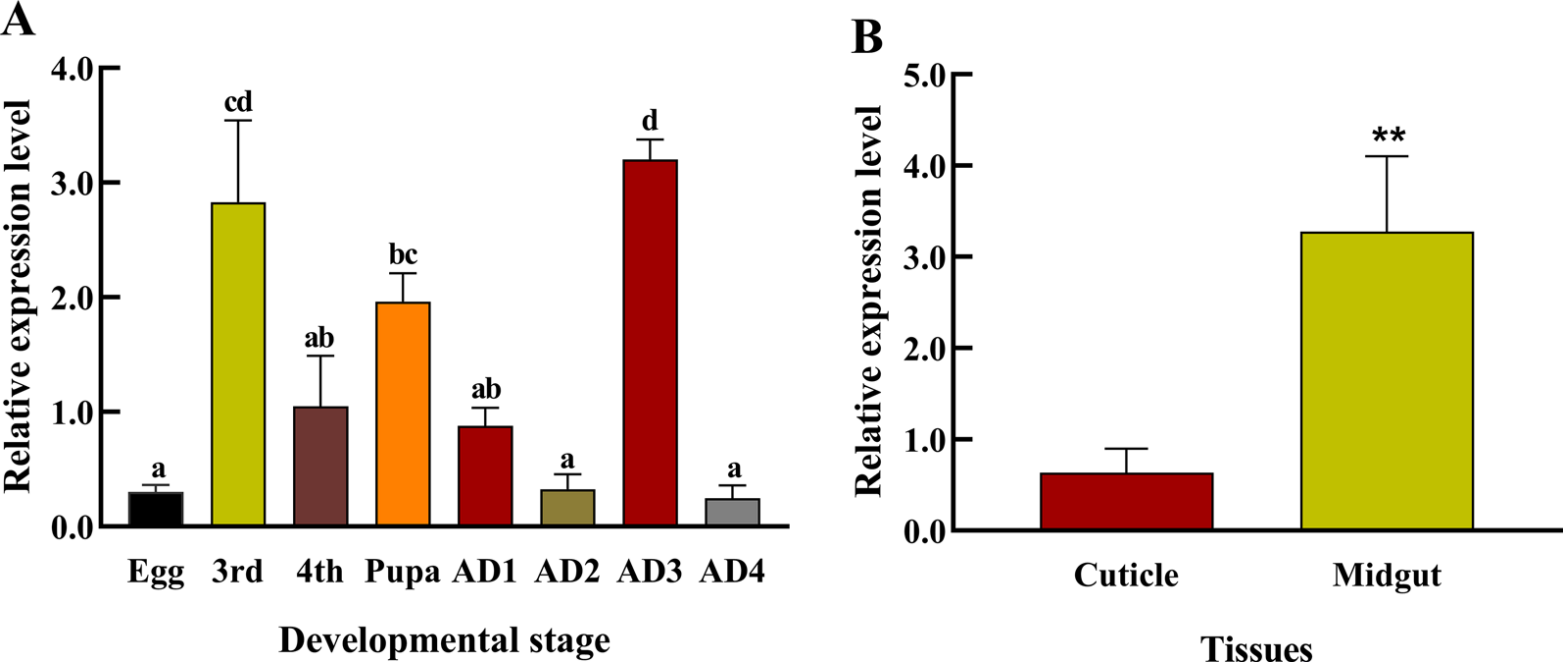
Expression patterns of *CHS-2* gene in *Ae. albopictus*. Real-time quantitative fluorescence PCR (qPCR) was used to detect the relative expression level of *CHS-2* in different periods of *Ae. albopictus* and in different tissues of the fourth-instar larvae of *Ae. albopictus*. **A** Relative expression of *CHS-2* in *Ae. albopictus* at different stages: egg, third-instar larva (3rd); fourth-instar larva (4th); adult *Ae. albopictus* (female) at 24 h after eclosion (AD1); adult *Ae. albopictus* (female) at 48 h after eclosion (AD2); adult *Ae. albopictus* (female) at 24 h after blood-sucking (AD3); adult *Ae. albopictus* (female) at 48 h after blood-sucking (AD4). Different lowercase letters above the bar indicate that the difference is statistically significant (one-way ANOVA, Tukey test, *P* < 0.05). **B** Relative expression of *CHS-2* in the midgut and cuticle of fourth-instar larvae. Relative expression levels were calculated in comparison with that at 4th (**A**) and cuticle (**B**), which were ascribed an arbitrary value of 1. Three biological replicates and three technical replicates were set. (One-way ANOVA, Duncan test, *P < 0.05, **P < 0.01)

The expression of *Ae. albopictus CHS-2* in the adult mosquitoes at 24 h after blood-sucking reached the highest value, followed by the third-instar larvae and pupal stages. After eclosion, the expression level of *CHS-2* in adult mosquitoes at 24 h and 48 h gradually decreased; the expression in adult mosquitoes at 48 h after blood-sucking was significantly decreased, and was almost at the same level as that in the initial egg stage (Fig. 2A). The expression of the *CHS-2* gene in the midgut was significantly higher than that in the epidermis in fourth-instar larvae (ANOVA, *F*_(1, 4)_ = 225.941, *P* = 0.004) (Fig. 2B).

### Evaluation of the silencing effect of *CHS-2*

**Fig. 3:**
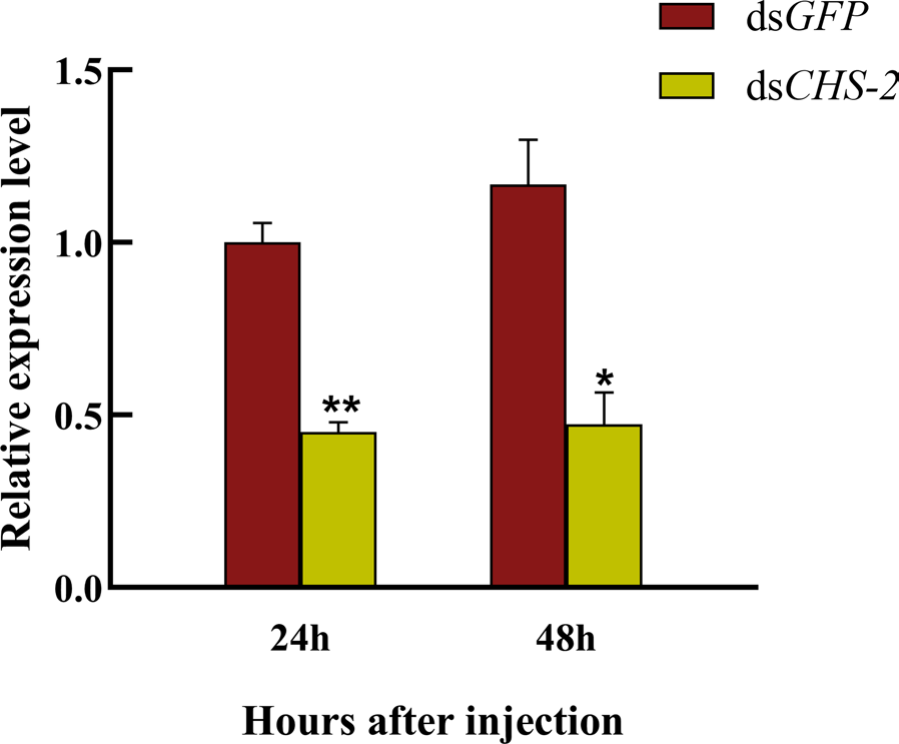
Detection of silencing efficiency. The relative expression of the *CHS-2* gene in *Ae. albopictus* larvae was detected by real-time quantitative PCR (qPCR) 24 h and 48 h after dsRNA injection. Relative expression levels were calculated in comparison with those at 24 h after ds*GFP* injection, which was ascribed an arbitrary value of 1. Three biological replicates and three technical replicates were set. (One-way ANOVA, Duncan test, *P < 0.05, **P < 0.01)

**Fig. 4:**
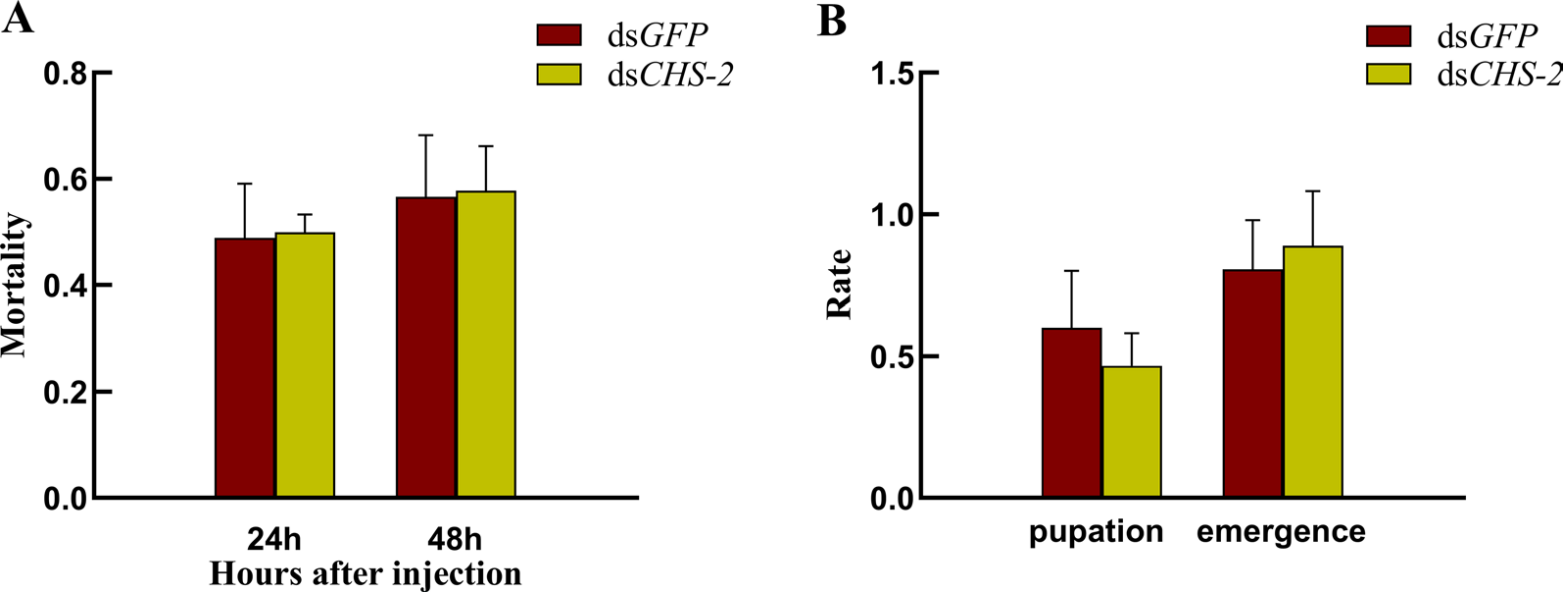
Survival and development of fourth-instar larvae of *Ae. albopictus* after *CHS-2* gene silencing. Fifty larvae of the fourth-instar *Ae. albopictus* were randomly selected and fed normally after injection of dsRNA. The mortality rate of larvae after 24 h and 48 h (**A**) and the pupation rate and emergence rate of larvae within 72 h (**B**) were recorded. Three biological replicates and three technical replicates were set. (One-way ANOVA, Duncan test)

After *Ae. albopictus* was injected with dsRNA, the expression of the *CHS-2* gene at 24 h after silencing was significantly downregulated compared to that in the control group (ANOVA, *F*_(1, 4)_ = 153.813, *P* = 0.006), and the expression at 48 h after silencing was also significantly downregulated (ANOVA, *F*_(1, 4)_ = 38.103, *P* = 0.025) (Fig. 3), indicating that the effect of RNA silencing was obvious, and the follow-up experiments could be carried out. *CHS-2* gene silencing had no effect on the survival of *Ae. albopictus* larvae (Fig. 4A). Pupation and emergence of larvae within 72 h after silencing were also unaffected (Fig. 4B).

### Changes in the expression of genes involved in the chitin synthesis pathway after *CHS-2* inhibition

**Fig. 5:**
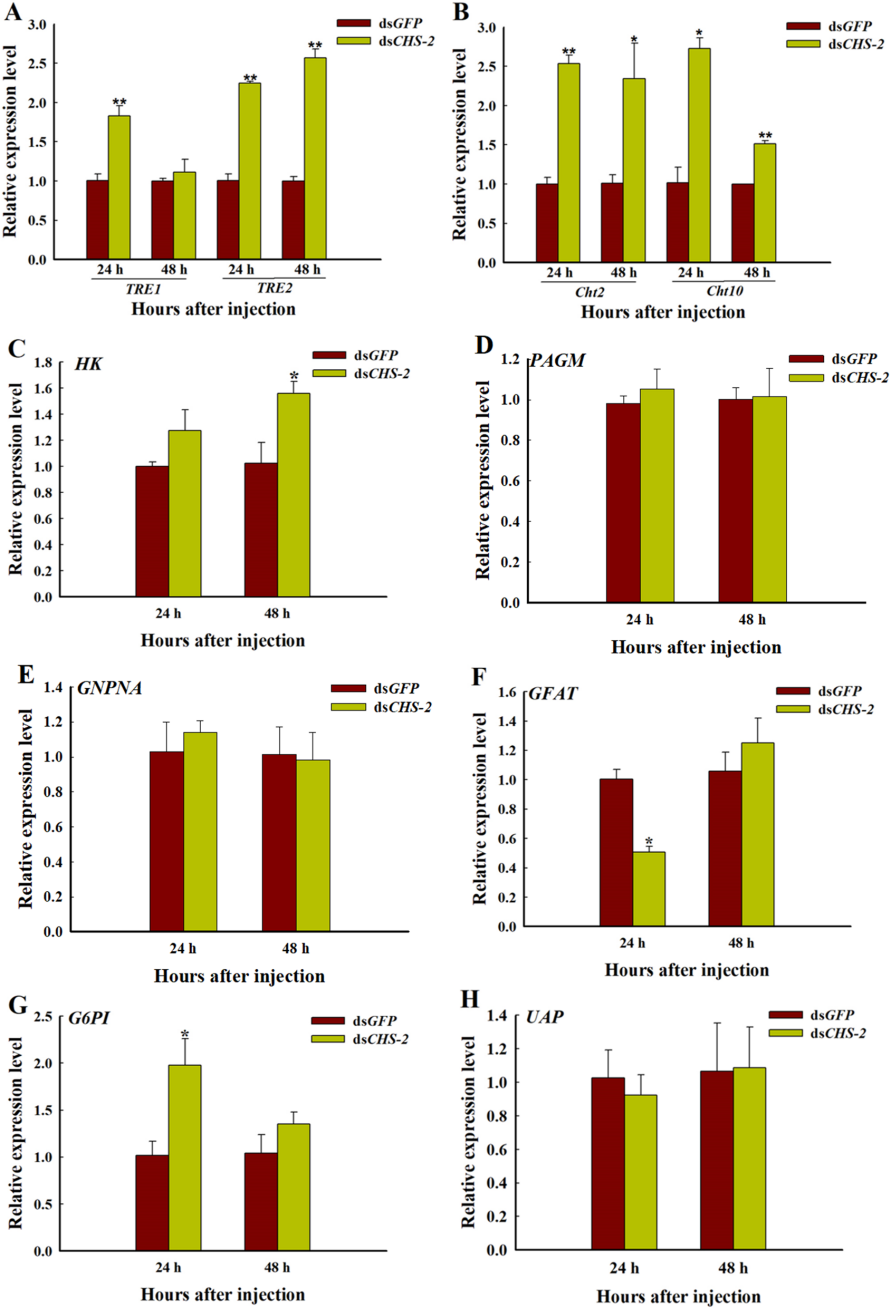
Expression of genes involved in chitin synthesis pathway in *Ae. albopictus* larvae after *CHS-2* silencing. **A** *TRE1* and *TRE2.* **B** *Cht2* and *Cht10.* **C** *HK*. **D** *PAGM*. **E** *GNPNA*. **F** *GFAT*. **G** *G6PI*. **H** *UAP*. Relative expression levels were calculated in comparison with those after ds*GFP* injection, which was assigned an arbitrary value of 1. Three biological replicates and three technical replicates were set. (One-way ANOVA, Duncan test, *P < 0.05, **P < 0.01)

Compared with the control group, the ds*CHS-2* treatment group exhibited significant upregulation of the chitin synthesis-related gene *TRE1* in *Ae. albopictus* (ANOVA, *F*_(1, 4)_ = 42.348*, P* = 0.003), and the recovery at 48 h was consistent with the control group (Fig. 5A). *TRE2* (ANOVA, *F*_(1, 4)_ = 66.4*, P* = 0.04) and chitin metabolism-related *Cht2* (ANOVA, *F*_(1, 4)_ = 90.11, *P* = 0.002) and *Cht10* (ANOVA, *F*_(1, 4)_ = 51.343,* P* = 0.019) were significantly upregulated at 24 h and 48 h (ANOVA, *TRE2 F*_(1, 4)_ = 92.384, *P* = 0.001, *Cht2 F*_(1, 4)_ = 8.294, *P* = 0.045, *Cht10 F*_(1, 4)_ = 112.28, *P* = 0.002) after injection (Fig. 5A, B). In addition, *HK* (ANOVA, *F*_(1, 4)_ = 8.414*, P* = 0.044) and *G6PI* (ANOVA, *F*_(1, 4)_ = 11.26*, P* = 0.044) gene expression were significantly upregulated after *CHS-2* silencing (Fig. 5C, G). Among the remaining genes, *PAGM*, *UAP*, and *GNPNA* showed no significant change (Fig. 5D, E, H); only *GFAT* (ANOVA, *F*_(1, 4)_ = 29.025*, P* = 0.013) expression was significantly downregulated 24 h after silencing (Fig. 5F).

### Changes in chitin levels and enzyme activity after *CHS-2* inhibition

**Fig. 6:**
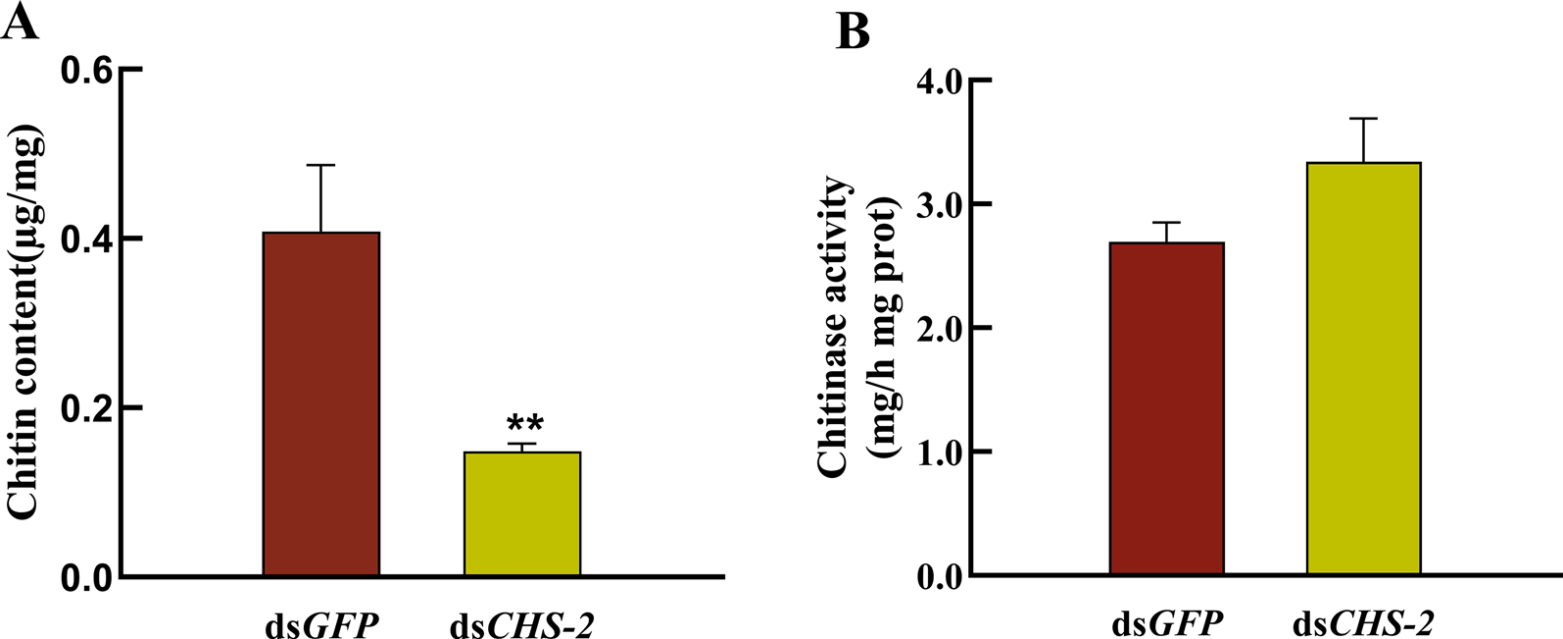
Changes of chitin content and enzyme activity in the midgut of *Ae. albopictus* larvae after *CHS-2* silencing. The fourth-instar larvae were dissected and the midguts of 45 larvae were collected at 24 h and 48 h after the injection of dsRNA. The content of chitin in the midgut of the larvae was determined by chitinase, and the activity of chitinase in the midgut of the larvae was determined by a chitinase kit. **A** Chitin content in the midgut of the fourth-instar larvae of *Ae. albopictus* 24 h after dsRNA injection. **B** Chitinase activity in the midgut of the fourth-instar larvae of *Ae. albopictus* 24 h after dsRNA injection. Three biological replicates and three technical replicates were set. (One-way ANOVA, Duncan test, *P < 0.05, **P < 0.01)

At 24 h after injection of ds*CHS-2*, the chitin level in the midgut of *Ae. albopictus* was significantly reduced compared with the control (ANOVA, *F*_(1, 4)_ = 21.653*, P* = 0.043) (Fig. 6A). However, after inhibiting *CHS-2* for 24 h, there was no significant change in chitinase activity in the midgut of the fourth-instar larvae of *Ae. albopictus* (Fig. 6B).

### Morphological changes in the midgut after *CHS-2* inhibition

**Fig. 7:**
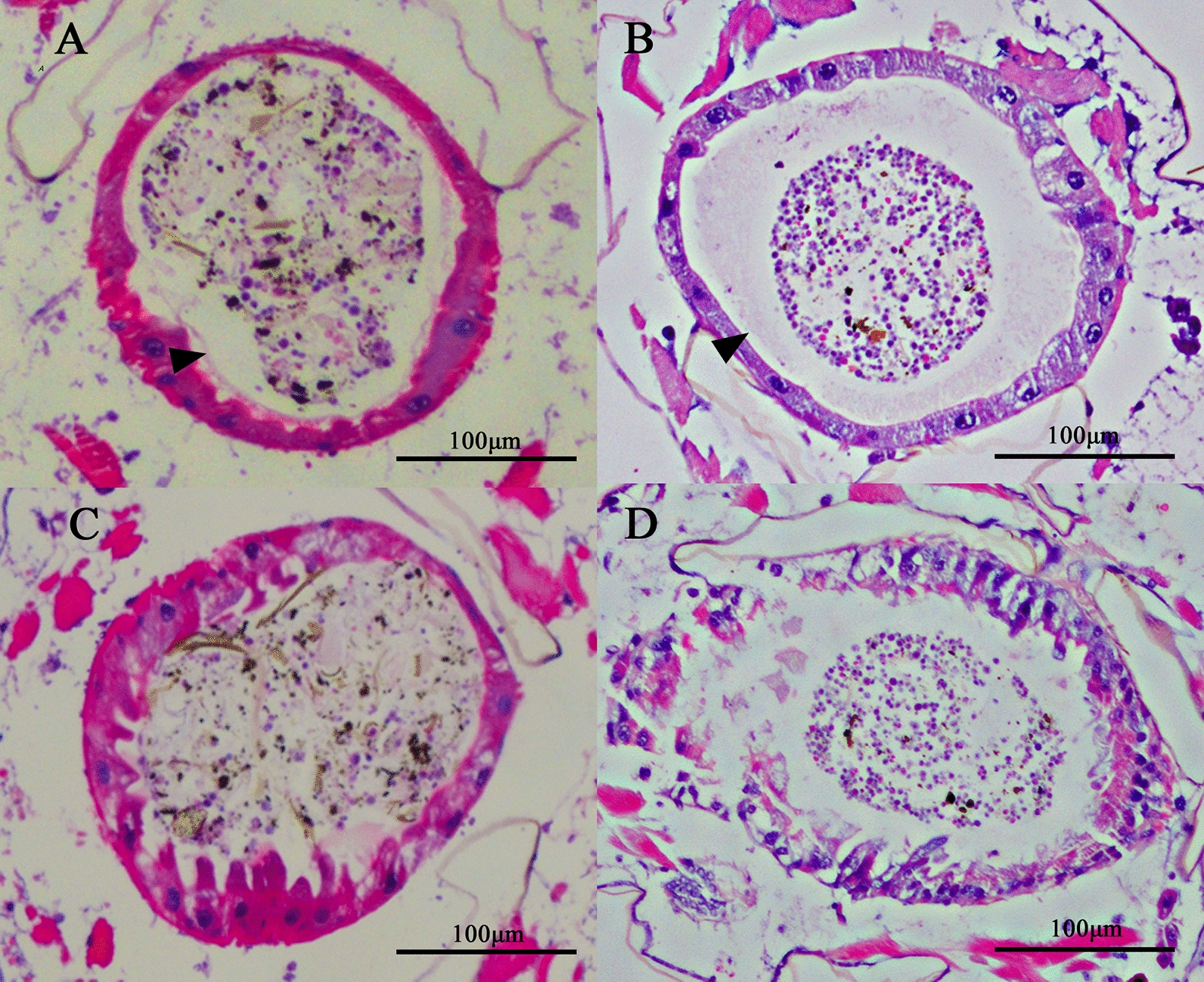
Section of fourth-instar larvae of *Ae. albopictus* after *CHS-2* silencing. Fourth-instar larvae 24 h and 48 h after injection of dsRNA were used as materials; the head and tail were removed, the larvae were embedded in paraffin, sectioned, and stained with HE, and they were observed with a microscope: **A** 24 h after the injection of ds*GFP*, **B** 48 h after the injection of ds*GFP*, **C** 24 h after the injection of ds*CHS-2*, **D** 48 h after the injection of ds*CHS-2*. Scale indicates 100 μm, the black arrow in the figure is the normal peritrophic membrane

At 24 h and 48 h after injection of ds*CHS-2*, the midgut epithelial cells of *Ae. albopictus* larvae showed vacuolization, intracellular convexity, and partial cell rupture, and the structure of the peritrophic membrane was destroyed or even absent. In the control group injected with ds*GFP*, the peritrophic membrane of the midgut was fully developed (Fig. 7).

## Discussion

Chitin synthase, the most important enzyme of the chitin synthesis pathway in insects, is a conserved enzyme present in all chitin-containing organisms. Tellam et al. first identified the gene sequence encoding insect chitin synthase in 2000 [54]. Since then, with the development of gene, protein, and transcriptome technologies, more genes that encode insect chitin synthetases have been discovered [55, 56]. Through multisequence alignment and evolutionary tree construction, it was found that the *CHS-2* protein of *Ae. albopictus* was highly conserved and was closely related to *Ae. aegypti*, belonging to the same genus, and clustered with the outgroup *Cx. quinquefasciatus* and *Anopheles coluzzii* (Fig. 1).

There are two kinds of CHS in insects, CHS-1 (A) and CHS-2 (B). Although the two share some similarities, they have different functions in insect growth and development. Previous studies have shown that CHS-1 (A) mainly functions in the insect epidermis and trachea, while CHS-2 (B) is mainly expressed in the insect midgut [57]. It has also been found that both proteins are expressed in the compound eyes of *An. gambiae* pupae [38]. At present, the insect *CHS-1* gene has been used as a potential target gene for pest control [58–61]. As research has progressed, the study of *CHS-2* as a potential target has also gradually gained substantial attention. For example, Lopez et al. and Singh et al. reported that feeding and exposing ds*CHS-2* to *Ae. aegypti* larvae both slowed larval development and attenuated stress resistance [5, 62]. In this study, we characterized the spatial and temporal expression pattern of *CHS-2* in *Ae. aegypti*. Its expression level was relatively high in the third-instar larva and pupal stage (Fig. 2A). This high expression was also found in *Cx. quinquefasciatus* and *An. gambiae* [38, 63]. Interestingly, the opposite pattern was found in several other insects [64]. Therefore, we speculate that *CHS-2* may play other roles besides the formation of mosquito pupae. The expression level reached the maximum at 24 h after blood-feeding, and significantly decreased at 48 h after blood-feeding, which was similar to the initial egg stage (Fig. 2A). The increase in *CHS-2* expression levels after feeding has been observed in other insects [65, 66], but the exact mechanism is not clear. We hypothesize that this upregulation may be because *Ae. albopictus* needs to form a large peritrophic membrane to digest blood. It may also be needed for reproduction by *Ae. albopictus*. Studies have shown that chitinoids are components of *Ae. aegypti* eggs [67], which needs to be investigated in subsequent experiments. Similar to other insects [57, 63], the *CHS-2* gene of *Ae. albopictus* also has obvious tissue expression specificity, being mainly expressed in the middle intestine and less expressed in the epidermis (Fig. 2B). Our studies and those of others have confirmed that *CHS-2* is directly involved in the synthesis of chitin in the midgut of insects and plays an important role in insect growth and development.

RNAi is described as posttranscriptional gene silencing, and serves as a research tool for not only gene function [68, 69], but also for developing RNA insecticides for sustainable pest management [70]. In this study, the *CHS-2* gene of *Ae. albopictus* was silenced by RNAi, the relative expression level was significantly downregulated, and the inhibitory effect was obvious (Fig. 3). *CHS-2* silencing had no significant influence on the survival of larvae and mature larvae (Fig. 4); the reason may be that the short duration of dsRNA action in microinjection or the possible impact of mechanical damage in microinjection on larval survival.

The expression of genes related to the chitin synthesis pathway was also measured. TRE is the first enzyme in the chitin synthesis pathway, and silencing *TRE* resulted in decreased expression levels of genes involved in chitin metabolism [71]. After knockdown of *CHS-2* for 24 h and 48 h, the expression of *TRE2* was significantly upregulated (Fig. 5A). Twenty-four hours after silencing, *TRE1* expression was significantly upregulated (Fig. 5A). HK and G6PI are also important enzymes in the chitin synthesis pathway [72, 73], and their expression levels were also significantly upregulated after *CHS-2* silencing (Fig. 5C, G). The above are all genes upstream of *CHS-2* in the chitin synthesis pathway, and after *CHS-2* is inhibited, these genes may be highly expressed in the short term to synthesize more chitin from other sites in response to the loss of chitin in the midgut. However, the expression of *GFAT* was significantly downregulated and returned to the same level as that of the control group at 48 h after silencing (Fig. 5F), which was consistent with the findings for *Spodoptera frugiperda* [52]. The specific mechanism needs further study. Pesch et al. found that the silencing of *Cht2* affected the level of chitin in the *Drosophila* midgut [74]. Zhu found that *Cht10* of *Plutella xylostella* was mainly involved in the process of moulting, pupation, and eclosion of larvae [75]. In this study, the expression of the two chitinase genes *Cht2* and *Cht10* was significantly or very significantly upregulated at 24 and 48 h after *CHS-2* silencing (Fig. 5B). However, the activity of chitinase in the midgut remained unchanged (Fig. 6B). It is speculated that one reason may be the time difference between gene expression and enzyme protein expression, and the other reason is that *CHS-2* silencing upregulates CHS expression in other parts of *Ae. albopictus* larvae aside from the midgut. *CHS-2* silencing caused a significant decrease in chitin levels in the larval midgut (Fig. 6A), consistent with other studies [62, 76]. In this study, samples of *Ae. albopictus* larvae after *CHS-2* silencing were collected for paraffin embedding, sectioning, and HE staining, and the results showed that the formation of the peritrophic membrane in the midgut of *Ae. albopictus* was affected after *CHS-2* silencing. The peritrophic membrane was thinner or even absent and the midgut epithelial cells were vacuolated (Fig. 7), which was similar to the results of previous studies [74, 77].

Overall, the results showed that *CHS-2* could affect the synthesis and degradation of chitin in the midgut of *Ae. albopictus* larvae, thus affecting the structure and function of the intestinal peritrophic membrane. The peritrophic membrane is a semipermeable tissue made of chitin and proteins that helps protect insects from pathogens and macromolecular compounds. Although silencing of *CHS-2* does not lead directly to larval death, broken peritrophic membranes may make larvae more sensitive to insecticides or heavy metal pollution. In a follow-up study in our laboratory (data not yet published), the mortality of *Ae. albopictus* larvae was significantly increased in response to cadmium stress after the *CHS-2* gene was silenced. Therefore, *CHS-2* represents a potential target for auxiliary control of *Ae. albopictus*, providing a theoretical basis for the population control of *Ae. albopictus*. Future research will focus on the following: first, delivery of dsRNA, where microinjection is obviously impractical, the exploration of more accessible silencing methods, such as nanocarriers [78], feeding [79], and gene editing [80, 81]. Further studies on the specific effects of *CHS-2* silencing on other physiological characteristics of *Ae. albopictus*, including maturation, reproduction, and stress resistance, are needed to facilitate the development of biocides targeting chitin synthesis and metabolism.

## Conclusions

We analysed the expression characteristics of the *CHS-2* gene in *Ae. albopictus*, and found that it was mainly expressed in the midgut, with the highest expression level in adult mosquitoes after blood-feeding. Compared with that in the midgut of larvae in the control group, the level of chitin in the midgut of larvae of the *CHS-2* knockdown group was significantly reduced, and the structure of the midgut peritrophic membrane was destroyed or even absent. Our results demonstrate that *CHS-2* is important for the formation of the peritrophic membrane in the midgut of *Ae. albopictus*. *CHS-2* gene silencing, although not directly affecting the survival of *Ae. albopictus*, can still be used to enhance the effects of other control methods, which may lead to a new direction for vector insect control and provide new ideas for research on reducing pest resistance.

## Data Availability

Data supporting the conclusions of this article are included within the article and its additional files. Raw data are available from the corresponding author upon request.

## Abbreviations

*CHS*: Chitin synthetase gene
*CHS-1*: Chitin synthase-1
*CHS-2*: Chitin synthase-2
BPU: Benzophenylurea
qPCR: Real-time fluorescence-based quantitative PCR
*TRE1*: Trehalase 1
*TRE2*: Trehalase 2
HK: Hexokinase
G6PI: Glucose-6-phosphate isomerase
GFAT: Glutamine–fructose-6-phosphate aminotransferase
GNPNA: Glucosamine-6-phosphate-*n*-acetyltransferase
PAGM: Phosphoacetylglucosamine mutase
UAP: Uridine diphosphate (UDP)-*N*-acetylglucosamine pyrophosphorylase
CHS: Chitin synthetase
CHSA: Chitin synthetase A
CHSB: Chitin synthetase B
RNAi: RNA interference
NCBI: National Center for Biotechnology Information
*GFP*: Green fluorescent protein gene
